# EZH2-mediated repression of GSK-3β and TP53 promotes Wnt/β-catenin signaling-dependent cell expansion in cervical carcinoma

**DOI:** 10.18632/oncotarget.8741

**Published:** 2016-04-15

**Authors:** Qian Chen, Peng-Sheng Zheng, Wen-Ting Yang

**Affiliations:** ^1^ Department of Reproductive Medicine, The First Affiliated Hospital of the Medical College, Xi'an Jiaotong University, Xi'an, The People's Republic of China; ^2^ Section of Cancer Stem Cell Research, Key Laboratory of Environment and Genes Related to Diseases, Ministry of Education of the People's Republic of China, Xi'an, The People's Republic of China

**Keywords:** EZH2, cervical cancer, proliferation, Wnt/β-catenin signaling, GSK3β and TP53

## Abstract

Enhancer of zeste homolog 2 (EZH2), a catalytic core component of the Polycomb repressive complex 2 (PRC2), stimulates the silencing of target genes through histone H3 lysine 27 trimethylation (H3K27me3). Recent findings have indicated EZH2 is involved in the development and progression of various human cancers. However, the exact mechanism of EZH2 in the promotion of cervical cancer is largely unknown. Here, we show that EZH2 expression gradually increases during the progression of cervical cancer. We identified a significant positive correlation between EZH2 expression and cell proliferation *in vitro* and tumor formation *in vivo* by the up-regulation or down-regulation of EZH2 using CRISPR-Cas9-mediated gene editing technology and shRNA in HeLa and SiHa cells. Further investigation indicated that EZH2 protein significantly accelerated the cell cycle transition from the G0/G1 to S phase. TOP/FOP-Flash reporter assay revealed that EZH2 significantly activated Wnt/β-catenin signaling and the target genes of Wnt/β-catenin pathway were up-regulated, including β-catenin, cyclin D1, and c-myc. Moreover, dual-luciferase reporter and chromatin immunoprecipitation (ChIP) assays confirmed that EZH2 inhibited the expression of glycogen synthase kinase-3β (GSK-3β) and TP53 through physically interacting with motifs in the promoters of the GSK-3β and TP53 genes. Additionally, blockage of the Wnt/β-catenin pathway resulted in significant inhibition of cell proliferation, and activation of the Wnt/β-catenin pathway resulted in significant enhancement of cell proliferation, as induced by EZH2. Taken together, our data demonstrate that EZH2 promotes cell proliferation and tumor formation in cervical cancer through activating the Wnt/β-catenin pathway by epigenetic silencing via GSK-3β and TP53.

## INTRODUCTION

Cervical cancer is the fourth most common tumor type and the fourth leading cause of cancer death among women worldwide, and its incidence has increased in recent years [1]. Nearly 90% of cervical cancer deaths have occurred in developing regions of the world. Thus, to develop better prognostic and therapeutic strategies for cervical cancer, in-depth research into the molecular and biologic mechanisms of oncogenesis is critical. It is known that although the development of cervical cancer is intimately associated with high-risk human papillomavirus (HPV) infection, progression of a HPV-positive premalignant lesion to invasive carcinoma is a rare event [2]. Thus, not all patients infected with HPV ultimately develop cervical cancer and various molecular abnormalities, including the inactivation of tumor suppressor genes and activation of oncogenes, which are also essential for cervical cancer development [3]. Extensive studies have shown that both genetic changes and epigenetic modifications may play important roles in complex signaling pathways in carcinogenesis [4–6], but the underlying mechanism of cervical cancer has not yet been clearly elucidated.

EZH2, a key component of the PRC2 complex, which is a transcriptional repressor that helps to maintain cell identity during development by promoting chromatin modifications [7], has been found to contribute to gene repression via the trimethylation of H3K27 [8] catalyzed by the SET domain of EZH2. EZH2 regulates several cellular processes, including cell fate determination, cell cycle regulation, senescence, cell differentiation and carcinogenesis [9]. Furthermore, EZH2 has been reported to be overexpressed and to function as an oncogene in various cancers by mediating the expression of target genes involved in tumorigenesis [9, 10], including prostate cancer [11], breast cancer [12, 13], hepatocellular carcinoma [14], colorectal cancer [15, 16], gastric cancer [17], ovarian cancer [18], melanoma [19] and cervical cancer [20–23], suggesting the potential role of EZH2 in tumor. Additionally, EZH2 has been found to act as an epigenetic modifier during the TGF-β-induced epithelial-mesenchymal transition (EMT) in breast carcinogenesis and to control the p38 mitogen-activated protein kinase (MAPK) signaling pathway during breast cancer cell migration, invasion and metastasis [24].

However, the molecular mechanisms of EZH2 in cervical carcinoma are largely unknown. In the present study, we demonstrated that EZH2 was up-regulated during the development and progression of cervical cancer and that the down-regulation of EZH2 using CRISPR-Cas9 knockout technology and shRNA in cervical cancer cells inhibited cell growth both *in vitro* and *in vivo* by inhibiting Wnt/β-catenin signaling, which has key roles in embryonic development [25] and many cellular processes, such as proliferation, migration, differentiation and apoptosis [25–27].

## RESULTS

### EZH2 expression in normal cervix and different cervical lesions

**Figure 1:**
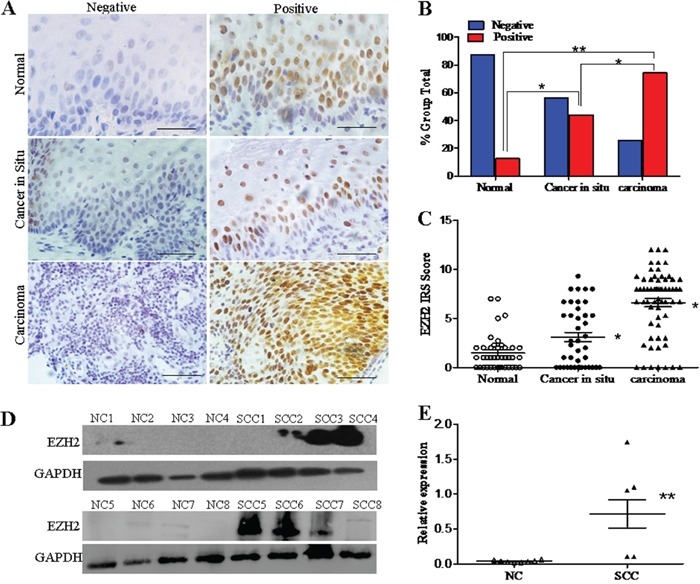
EZH2 expression in normal cervixes and different cervical lesions **A.** Immunohistochemistry results showing EZH2 expression in normal cervix, cervical carcinoma *in situ*, and cervical carcinoma tissues; scale bar, 50 μm. **B.** EZH2 staining is classified into 2 categories (negative and positive), and the percentage of tissues in each group is shown. **C.** The IHC scores of EZH2 staining in the normal cervix, carcinoma *in situ*, and carcinoma tissues are shown. **D.** Representative Western blots of EZH2 protein expression in NC and SCC. **E.** Quantitative analysis of EZH2 expression in normal cervical and squamous cervical carcinoma tissues; GAPDH was used as an internal control, and Student's t-test was carried out. * *p<0.05*, ** *p<0.01*.

To explore the function of EZH2 in the development and progression of cervical carcinoma, immunohistochemistry was first conducted using paraffin-embedded normal cervix (NC), cervical carcinoma *in situ* (CIS), and cervical carcinoma (CC) tissues. EZH2 staining was observed in the nuclei of positive cells in all of the different cervical tissues (Figure 1A). The number of specimens with positive EZH2 staining gradually increased from 12.5% (5/40) in the normal cervical tissues to 43.9% (18/41) in the cervical cancer *in situ* tissues, and then to 74.2% (46/62) in the cervical cancer tissues (Supplementary Table 1 and Figure 1B). Analysis of the IHC scores also revealed that the immunoreactivity score (IRS) of EZH2 staining was 1.5 for the normal cervical tissues, 3.1 for the CIS tissues and 6.6 for the cervical cancer tissues (*p*<0.05, Figure 1C).

Furthermore, EZH2 protein expression was detected by western blotting in 8 normal cervical specimens and 8 primary cervical carcinoma specimens (Figure 1D). The average relative EZH2 expression level was 0.71 in the cervical cancer tissues and 0.04 in the normal cervical tissues. The cervical cancer tissues had a 17-fold higher EZH2 expression level than the normal cervical tissues (Figure 1E, *p*<0.01). These results suggest that EZH2 is highly expressed in cervical cancer and that it may promote the development and progression of cervical carcinoma.

### EZH2 promotes tumor formation of cervical cancer cells *in vivo*

**Figure 2:**
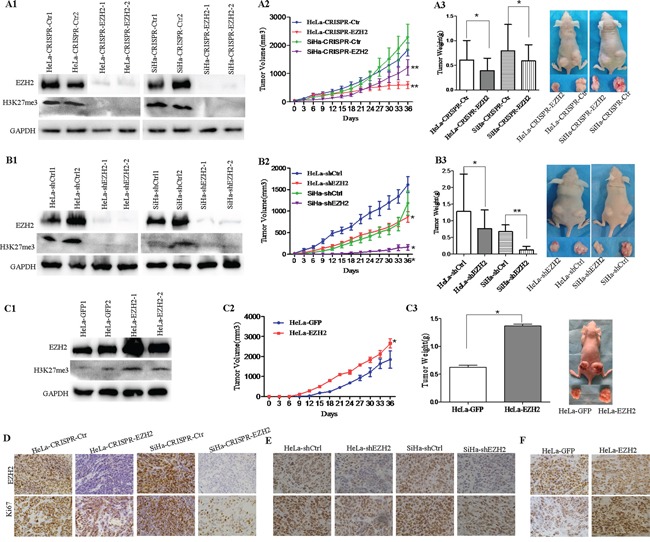
Depletion of EZH2 suppresses the growth of cervical cancer xenografts *in vivo* **A1-A3.** Tumor growth curves and tumor weights are shown for CRISPR/Cas9-mediated EZH2-knockout HeLa (HeLa-CRISPR-EZH2) cells, SiHa (SiHa-CRISPR-EZH2) cells and control cells (HeLa-CRISPR-Ctr, SiHa-CRISPR-Ctr). **B1-B3.** Tumor growth curves and tumor weights are shown for EZH2-knockdown HeLa (HeLa-shEZH2) cells, SiHa (SiHa-shEZH2) cells and control cells (HeLa-shCtrl, SiHa-shCtrl). **C1-C3.** Tumor growth curves and tumor weights are shown for EZH2-overexpressing HeLa (HeLa-EZH2) cells and HeLa-GFP cells. Immunohistochemical staining results for EZH2 and Ki67 are shown in tumor xenografts of CRISPR-mediated EZH2-silenced HeLa and SiHa cells **D.**, HeLa-shEZH2 and SiHa-shEZH2 cells **E.** and EZH2-overexpressing HeLa cells **F.** The values are presented as the mean±SD. * *p<0.05*, ** *p<0.01*.

Next, using immunohistochemistry and western blot assay, we found that EZH2 showed different expression levels in five cervical cancer cell lines, including HeLa, SiHa, C33A, CaSki and HT-3 (Supplementary Figure 1). To further investigate the role of EZH2 in cervical carcinogenesis, we employed CRISPR/Cas9 technology targeting exon 2 of EZH2 and identified genetic interruption and significant down-regulation of the EZH2 protein in HeLa and SiHa cell lines (Figure 2A1). Additionally, EZH2 down-regulation in cervical cancer cells silenced by shRNA was also observed in HeLa and SiHa cells (Figure 2B1), H3K27me3 levels were also obviously decreased.

To determine whether EZH2 affects tumor formation ability, female nude mice (6 mice per group) were injected subcutaneously with EZH2-modified cervical cancer cells. Tumor growth was monitored in terms of tumor volume every three days, and the net weights of the sacrificed mice were recorded upon termination of the experiment. The results showed that HeLa-CRISPR-EZH2 and HeLa-shEZH2 cells formed tumors that were much smaller (Figure 2A2 and 2B2; P<0.01) and lighter (Figure 2A3 and 2B3; P<0.05) than those formed by HeLa-CRISPR-Ctr and HeLa-shCtrl cells, respectively. Similarly, the tumors from SiHa-CRISPR-EZH2 and SiHa-shEZH2 cells were also smaller (Figure 2A2 and 2B2; P<0.01) and lighter (Figure 2A3 and 2B3; P<0.05, P<0.01) than those from SiHa-CRISPR-Ctr and SiHa-shCtrl cells, respectively. Furthermore, xenograft assay using stable EZH2-overexpressing HeLa cells showed that the tumors formed by HeLa-EZH2 cells appeared earlier (a palpable tumor formed at 9 days for HaLa-EZH2 cells and at 12 days for HeLa-GFP cells), progressed much faster and were heavier than those formed by HeLa-GFP cells (Figure 2C2 and 2C3, P<0.05). These results indicate that the EZH2 protein promotes tumor formation from cervical cancer cells *in vivo*.

To determine whether the tumor promotion of EZH2 is involved in cell proliferation, the expression of Ki67, a well-known cell proliferation marker, was examined in EZH2-modified cervical cancer cells from tumor xenograft tissues and in control cells by immunohistochemical staining. Both Ki67 and EZH2 staining were weaker in the xenograft tissues formed by EZH2-silenced HeLa and SiHa cells than those formed by control cells, respectively (Figure 2D and 2E, Supplementary Figure 3). Furthermore, tumor tissues formed by EZH2-overexpressing HeLa cells expressed much more EZH2 protein, stronger Ki67 staining and much more Ki67-positive cells (Figure 2F, Supplementary Figure 3). These results suggest that EZH2 promotes tumor formation in cervical carcinoma, possibly by enhancing the proliferation of cervical cancer cells.

### EZH2 promotes the proliferation of cervical cancer cells by accelerating the cell cycle transition from G0/G1 to S phase

**Figure 3:**
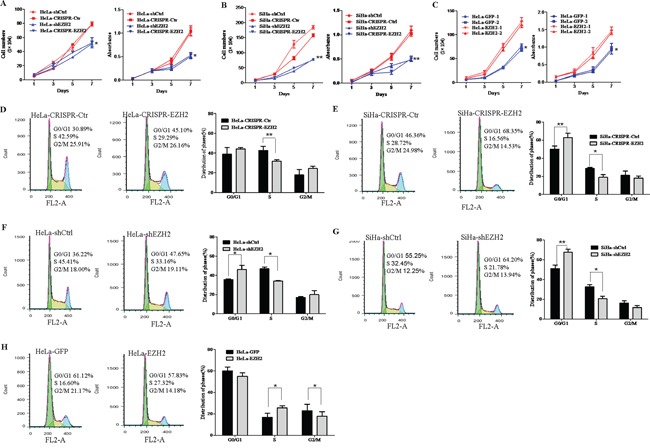
EZH2 promotes the proliferation of cervical cancer cells by accelerating the cell cycle transition from G0/G1 to S phase Cell growth and MTT assays were performed using EZH2-depleted HeLa cells **A.** EZH2-depleted SiHa cells **B.** and EZH2-overexpressing HeLa cells **C.** Cell cycle progression was analyzed by FACS analysis, and the results of quantitative analysis of cell cycle distribution are shown for EZH2-silenced SiHa and HeLa cells **D-G.** and EZH2-overexpressing HeLa cells **H.** The values are presented as the mean±SD. * *p*<0.05, ** *p*<0.01.

Next, HeLa and SiHa cells with knock down of EZH2, as mediated by both CRISPR/Cas9 (HeLa-CRISPR-EZH2 and SiHa-CRISPR-EZH2) and shEZH2 (HeLa-shEZH2 and SiHa-shEZH2), showed significantly reduced cell growth and viability than the control cells (HeLa-CRISPR-Ctr, SiHa-CRISPR-Ctr, HeLa-shCtrl and SiHa-shCtrl), as determined by cell growth curve assay and MTT assay (Figure 3A and 3B, P<0.05). Meanwhile, overexpression of EZH2 in HeLa cells resulted in significant increases in cell growth and viability (Figure 3C). These results demonstrate that EZH2 enhances the proliferation of cervical cancer cells *in vitro*.

To investigate the mechanism of the EZH2 protein-mediated enhancement of cervical cancer cell proliferation, cell cycle analysis of EZH2-modified HeLa and SiHa cells and control cells was performed by fluorescence-activated cell sorting (FACS). The percentage of S phase cells significantly decreased from 42.7% for HeLa-CRISPR-ctrl cells to 31.6% for HeLa-CRISPR-EZH2 cells (Figure 3D, P<0.01), suggesting that EZH2 silencing induced HeLa cell cycle arrest in the G1/S-phase transition. A similar effect was observed in SiHa-CRISPR-EZH2 cells, of which 18.8% were in the S phase compared with 28.7% of control cells (Figure 3E, P<0.05). Next, analysis of cell cycle using shEZH2-transfected HeLa and SiHa cells was performed, showing the same results (Figure 3F and 3G). However, much more of the EZH2-overexpressing HeLa cells were in S phase compared with the control GFP cells (Figure 3H, P<0.05), suggesting that overexpression of EZH2 promoted the transition of the HeLa cells from G0/G1 phase to S phase. Collectively, these results suggest that EZH2 may accelerate the cell cycle transition at the G1/S phase and further promote the proliferation of cervical cancer cells.

### EZH2 activates the Wnt/β-catenin pathway in cervical carcinogenesis

**Figure 4:**
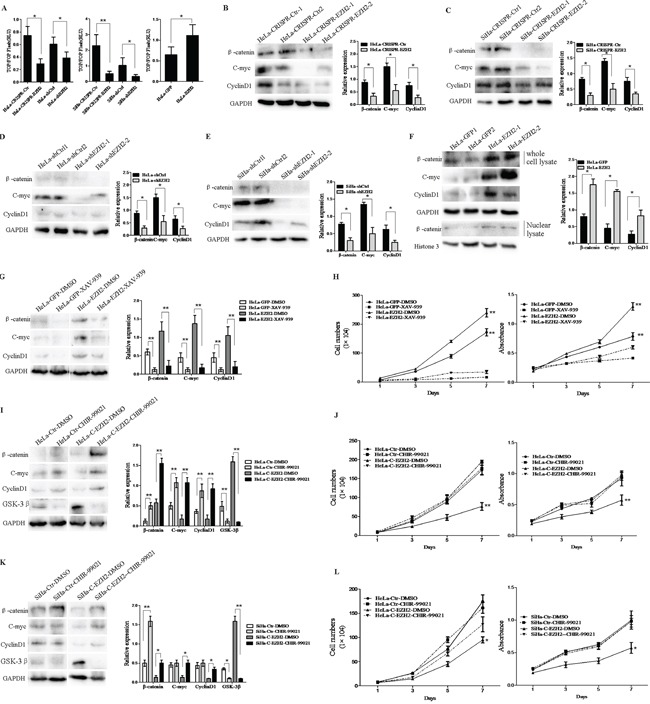
Depletion of EZH2 decreases activity of the Wnt/β-catenin pathway **A.** EZH2-depleted and EZH2-overexpessing HeLa and SiHa cells were transfected with a TOP/FOP-Flash reporter plasmid, and reporter activities were detected at 48 h after transfection by luciferase assay. **B-E.** The expression of β-catenin, c-myc, and cyclin D1 (Wnt/β-catenin pathway target genes) in EZH2-depleted HeLa and SiHa cells was determined by western blotting, including the quantitative analysis of β-catenin, c-myc, and cyclinD1 expression. **F.** The expression of β-catenin, c-myc, and cyclinD1 in EZH2-overexpressing HeLa cells were determined by western blot, including the quantitative analysis of β-catenin, c-myc, and cyclinD1 expression. **G.** EZH2-overexpressing HeLa cells were treated with a β-catenin inhibitor, XAV-939, and the expression of β-catenin, c-myc, and cyclin D1 was measured by western blotting. **H.** The effects of XAV-939 on the proliferation and viability of EZH2-overexpressing HeLa cells was evaluated by cell counting and MTT. **I** and **K.** The representative western blot and the quantitative analysis of β-catenin, c-myc, cyclin D1 and GSK-3β expression are shown following treatment with a GSK-3β inhibitor, CHIR-99021, for EZH2-knockout HeLa cells and SiHa cells. **J** and **L.** The effects of CHIR-99021 on proliferation and viability were evaluated. All data are the mean±SD from three independent experiments. *p <0.05, **p<0.01.

A previous study has described EZH2-activated Wnt/β-catenin signaling in gastric cancer [28] and hepatocellular carcinoma [29]. However, the mechanisms of the EZH2-mediated promotion of the proliferation and tumor formation of cervical cancer cells have not been shown to involve the activation of Wnt/β-catenin signaling. Thus, TOP/FOP flash luciferase reporter assay was used to examine the effect of EZH2 on β-catenin/TCF-dependent transcriptional activity, as a canonical experiment for the detection of Wnt/β-catenin signaling activity [30] (Figure 4A). The TOP/FOP flash reporter activities in HeLa and SiHa cells with the silencing of EZH2 by both CRISPR/Cas9 and shRNA were significantly inhibited (HeLa, *p*<0.05; SiHa, *p*<0.01). However, the luciferase signal was increased by approximately 2-fold in the HeLa-EZH2 cells compared with the control cells (*p*<0.05). These results suggest that EZH2 expression is positively related to the activity of the Wnt/β-catenin pathway in cervical cancer.

Next, the expression of β-catenin and the target genes of the Wnt/β-catenin pathway, including cyclin D1 and c-myc, were detected by western blotting using EZH2-silenced HeLa and SiHa cells and control cells (Figure 4B-4E). The relative expression levels of these proteins normalized by GAPDH expression were all decreased in the EZH2-silenced HeLa and SiHa cells compared with the control cells (*p*<0.05). Consistent with the activation of Wnt/β-catenin signaling, the expression of the β-catenin, cyclin D1, and c-myc proteins in the EZH2-overexpressing HeLa cells was significantly increased compared with that in the control cells (*p*<0.05). However, the accumulation of β-catenin in cytoplasm and its translocation into the nucleus consistently occurred following activation of the Wnt/β-catenin pathway, leading to the transcription of downstream target genes [26]. The expression level of β-catenin in the nuclei of EZH2-overexpressing HeLa cells was higher than that in the nuclei of HeLa-GFP cells, as determined by western blotting (Figure 4F). These results demonstrate that EZH2 expression activates the Wnt/β-catenin pathway, with increased expression of the target genes cyclin D1 and c-myc, in cervical cancer cells.

To further confirm that the Wnt/β-catenin pathway is the pathway by which EZH2 promotes the proliferation of cervical cancer cells, XAV939, which is an inhibitor of Wnt/β-catenin that acts by stimulating β-catenin degradation via stabilizing axin, a component of the β-catenin destruction complex [31], and CHIR-99021, which is an inhibitor of GSK-3β that suppresses β-catenin degradation [32], were used to activate or block the Wnt/β-catenin pathway in EZH2-modified HeLa and SiHa cells. When the HeLa-EZH2 cells were treated with XAV939, the protein levels of β-catenin, cyclin D1 and c-myc were significantly decreased compared to those in the cells treated with DMSO (Figure 4G). Meanwhile, XAV939 caused significant inhibition of cell proliferation and viability in the EZH2-overexpressing HeLa cells (*p*<0.01, Figure 4H). In contrast, the protein levels of β-catenin, cyclin D1 and c-myc in the CHIR-99021-treated EZH2-knockout HeLa and SiHa cells were significantly increased compared to those in the DMSO-treated cells (Figure 4I and 4K). Consistent with the above observations, CHIR-99021 treatment resulted in significant enhancements in cell proliferation and viability in the EZH2-knockout HeLa and SiHa cells (HeLa *p*<0.01, Figure 4J; SiHa *p*<0.05, Figure 4L). Taken together, these results demonstrate that the EZH2-mediated promotion of cervical cancer cell proliferation is regulated by activation of the Wnt/β-catenin pathway.

### EZH2 activates the Wnt/β-catenin pathway through direct transcriptional silencing of the expression of GSK-3β and TP53 in cervical cancer cells

**Figure 5:**
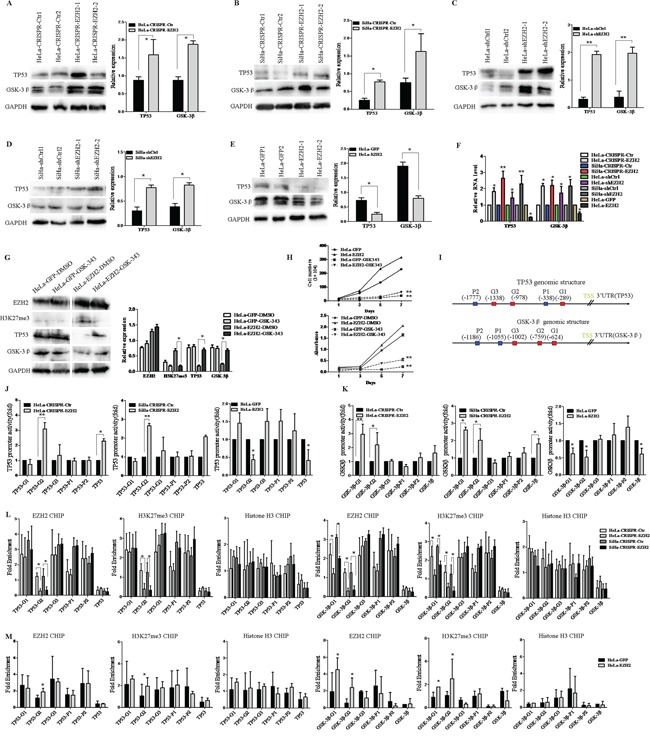
EZH2 activates the Wnt/β-catenin pathway through direct transcriptional repression of the expression of GSK-3β and TP53 in cervical cancer cells **A-D.** The expression of GSK-3β and TP53 in EZH2-depleted HeLa and SiHa cells was determined by western blotting. **E.** The expression of GSK-3β and TP53 in EZH2-overexpressing HeLa cells was determined by western blotting. **F.** The relative mRNA levels of TP53 and GSK-3βin EZH2-modified HeLa and SiHa cells as determined by quantitative real-time–PCR. **G.** The representative western blot and the quantitative analysis of EZH2, H3K27me3, TP53 and GSK-3β expression are shown following treatment with a EZH2 methyltransferase inhibitor, GSK-343, for EZH2-overexpressing HeLa cells. **H.** The effects of GSK-343 on proliferation and viability were evaluated. **I.** The GSK-3β and TP53 promoter structures containing the putative Polycomb response element (PRE), including the PHO and GAGA motifs, are shown. **J** and **K.** TP53 and GSK-3β promoter activities in EZH2-knockout and EZH2-overexpressing HeLa and SiHa cells. Luciferase activity relative to Renilla control activity was measured. **L** and **M.** The qChIP assay results are shown, with EZH2, H3K27me3 and H3 co-occupying the TP53 and GSK-3β promoter regions in EZH2-knockout and EZH2-overexpressing HeLa and SiHa cells. Immunoglobulin G (IgG) was used as a negative control. All data are the mean±SD from three independent experiments. *p<0.05, **p<0.01.

We next elucidated the mechanism whereby EZH2 activates the Wnt/β-catenin pathway in cervical cancer. EZH2 is known to be a key component of the PRC2 complex, which functions as a transcriptional inhibitor by binding to target genes [33, 34]. On the other hand, GSK-3β and TP53 are considered to play negative roles in this pathway by promoting the degradation of β-catenin [35, 29, 36]. Interestingly, the relative expression levels of the GSK-3β and TP53 proteins normalized by GAPDH were both increased in EZH2-silenced HeLa and SiHa cells, as determined by western blot analysis (Figure 5A-5D). In contrast, the expression of GSK-3β and TP53 proteins was dramatically inhibited after ectopic expression of EZH2 in HeLa cells (Figure 5E). Next, the mRNA levels of GSK-3β and TP53 were examined by Real-Time PCR in EZH2-modified HeLa and SiHa cells. Correspondingly, the mRNA levels of GSK-3β and TP53 normalized by β-actin were both increased in EZH2-silenced HeLa and SiHa cells (Figure 5F). The mRNA levels of GSK-3β and TP53 were decreased in EZH2-overexpressing HeLa cells (Figure 5F). To determine whether the effect of EZH2 on GSK3β and TP53 depend on the methyltransferase activity, GSK-343, which is a specific inhibitor of EZH2 methyltransferase, was used to block the methyltransferase activity of EZH2 in EZH2-overexpressing HeLa cells. When the HeLa-EZH2 cells were treated with GSK-343, the protein levels of GSK3β and TP53 were significantly increased compared to those in the cells treated with DMSO (Figure 5G). Meanwhile, GSK-343 caused significant inhibition of cell proliferation and viability in the EZH2-overexpressing HeLa cells (*p*<0.01, Figure 5H). These findings might suggest that EZH2 activates the Wnt/β-catenin pathway through the inhibition of GSK-3β and TP53 depending on the methyltransferase activity, which might be novel targets of EZH2 in cervical cells. To confirm this hypothesis, we performed dual-luciferase reporter assay to determine whether EZH2 represses the promoter activities of GSK-3β and TP53 by constructing GSK-3β and TP53 promoter-luciferase reporters containing the putative Polycomb response element (PRE), including the PHO and GAGA motifs (Supplementary Table 2), which can be co-occupied by EZH2 and H3K27me3 [37] (Figure 5I). The results showed that the luciferase activities of GSK-3β and TP53 in EZH2-knockdown HeLa and SiHa cells were more than two times higher than those in control cells at the TP53-G2 and GSK-3β-G1 and G2 positions. The luciferase activities of GSK-3β and TP53 in HeLa-EZH2 cells were more than two times lower than those in HeLa-GFP cells at the TP53-G2 and GSK-3β-G1 and G2 positions (Figure 5J and 5K). These results indicate that EZH2 transrepresses the expression of GSK-3β and TP53 in cervical cancer cells.

Furthermore, we performed quantitative ChIP (qChIP) assay to determine whether the EZH2 protein binds to specific regions of the GSK-3β and TP53 promoters *in vivo*. Five pairs of primers were designed to amplify the promoters of GSK-3β and TP53. Immunoprecipitation using a EZH2 antibody revealed the enrichment of EZH2, with binding to the promoters at the TP53-G2 and GSK-3β-G1 and G2 positions (Figure 5L and 5M). These results suggest that EZH2 specifically binds to the GSK-3β and TP53 promoters in cervical cancer cells. EZH2 has been confirmed to down-regulate target genes, as mediated by the demethylation of lysine 27 histone H3 (H3K27) [38, 39]. In support of this finding, q-ChIP assay revealed enrichment of the H3K27 trimethylation protein, which could also bind to the promoters (Figure 5L and 5M, Supplementary Table 3). Taken together, our findings suggest that EZH2 down-regulates the expression of GSK-3β and TP53 by specifically and directly binding to the promoters of GSK-3β and TP53 via histone methylation in cervical cancer cells.

### Correlation analysis between the expression of EZH2 and β-catenin, Cyclin D1, c-myc, GSK-3β and TP53 in cervical cancer specimens

**Figure 6:**
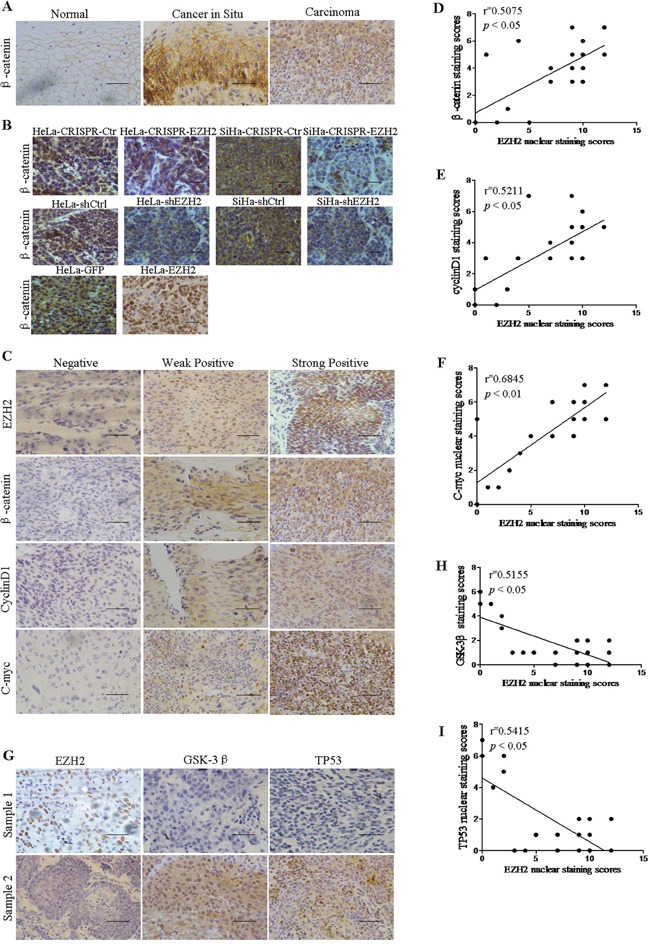
EZH2 expression is positively correlated with the expression of Wnt signaling-related proteins and negatively correlated with the expression of GSK-3β and TP53 in human cervical cancer tissues **A.** Immunohistochemistry results showing β-catenin expression in normal cervix, cervical carcinoma *in situ*, and cervical carcinoma tissues. **B.** Immunohistochemical staining results for β-catenin are shown in tumor xenografts of CRISPR-mediated EZH2-silenced HeLa and SiHa cells, HeLa-shEZH2 and SiHa-shEZH2 cells and EZH2-overexpressing HeLa cells. **C.** The expression of EZH2, β-catenin, c-myc and cyclin D1 were detected in 24 cervical cancer specimens by immunohistochemical staining. The correlation between the nuclear staining of EZH2 and β-catenin (**D.** r=0.5075; *P*<0.05), cyclin D1 (**E.**, r=0.5211; *P*<0.05) and c-myc (**F.**, r=0.6845; *P*<0.01) were significant, as determined by Pearson's correlation test. **G.** The expression of EZH2, GSK-3β and TP53 was detected in 24 cervical cancer specimens by immunohistochemical staining. **H** and **I.** The correlation of the nuclear staining of EZH2 and GSK-3β (r=0.5155; *P*<0.05) and TP53 (r=0.5415; *P*<0.05) were significant. All data are the mean±SD from three independent experiments. *p<0.05. **p<0.01.

To validate the correlation between the expression of EZH2 and Wnt/β-catenin signaling-related proteins in cervical cancer specimens, EZH2, β-catenin, cyclin D1, and c-myc expression levels were detected by IHC staining (Figure 6A, 6B). Notably, the β-catenin protein was localized to the membranes of normal cervical epithelial cells, and it was accumulated in the cytoplasm and nucleus in cervical cancer cells (Figure 6A). Also, β-catenin protein was localized to nucleus in xenograft tissues formed by EZH2-overexpressing HeLa cells and to membranes or no staining in other tumor tissues formed by EZH2-depleted HeLa and SiHa cells and control cells by immunohistochemical staining (Figure 6B). These results suggested that the staining pattern of β-catenin was likely consistent with the progression of normal cervical tissue to cancer. Furthermore, we found that EZH2 expression was significantly positively correlated with β-catenin, cyclin D1, and c-myc expression in 24 randomly selected cervical cancer samples (Figure 6D-6F). Therefore, these results support the hypothesis that EZH2 is associated with increased activity of the Wnt/β-catenin pathway in cervical cancer. Additionally, the expression of EZH2 was negatively correlated with the expression of GSK-3β and TP53 in cervical carcinoma tissues, as determined by immunohistochemistry (Figure 6G-6I). These results support the function of EZH2 as a negative regulator of GSK-3β and TP53 in cervical cancer tissues.

## DISCUSSION

The overexpression of EZH2 was first found to be associated with carcinogenesis in prostate cancer [11] and breast cancer [40] by microarray studies. Subsequently, elevated expression of EZH2 has already been demonstrated to promote cancer cell proliferation in multiple cancers, including bladder cancer [41], lung cancer [42], hepatocellular carcinoma [43] and gastric cancer [44]. Further, EZH2 KD was shown to induce a G2/M arrest in breast cancer and regulated cyclin D1, and β-catenin [45, 46]. P53 was shown to regulate EZH2 in undifferentiated nasopharyngeal carcinoma [47] and endometrial carcinoma [48]. However, recent studies have identified acquired EZH2 mutations in lymphoma cancer [49], myeloid neoplasms [50] and melanoma that are associated with the development of malignancy by regulating the expression of the complex of PcG genes to determine cell fate [9, 19, 51].

In our study, EZH2 expression was found to be significantly and gradually increased from normal cervical tissues to cervical cancer *in situ* tissues and then to cervical cancer tissues (Figure 1), in agreement with the results of previous studies of cervical cancer [20], prostate cancer [11] and breast carcinoma [52]. Subsequently, to further explore the function of EZH2 in cervical carcinogenesis, cervical cancer cells with exogenous or disrupted EZH2 were obtained in cervical cancer cell lines HeLa and SiHa. The results showed that EZH2 significantly promoted tumor growth *in vivo* and the proliferation of cervical cancer cells *in vitro* by accelerating the cell cycle transition of cervical cancer cells from G0/G1 phase to S phase (Figure 2 and 3).

The canonical Wnt/β-catenin signaling pathway, which activates the ability of the β-catenin protein to transactivate specific target genes, has been reported to be associated with various human diseases, including human carcinomas [53, 54]. Here, we confirmed that the Wnt/β-catenin pathway was stimulated by EZH2 in HeLa and SiHa cells, with activation of target genes, resulting in the promotion of cell growth and tumor formation (Figure 4). Additionally, EZH2-stimulated epigenetic silencing contributes to constitutive activation of Wnt/β-catenin signaling and consequential cellular proliferation in hepatocellular carcinoma [55], gastric cancer [28], renal cell carcinoma [56], colon cancer [57] and breast cancer [58]. In the mechanism of the EZH2-mediated activation of Wnt/β-catenin signaling to promote carcinogenesis, EZH2, which acts as a transcriptional repressor, represses Wnt antagonists, including RAF1, CXXC4, AXIN2/betaTrCP and p53 [13, 28, 55, 59], by decreasing the acetylation of H3K27, thereby promoting the development of different types of cancers. However, in breast cancer, EZH2 acts as a dual-function transcriptional regulator with dynamic activity by transactivating estrogen and Wnt pathways via physically interacting directly with estrogen receptor and β-catenin to promote cell cycle progression [60].

In the present study, luciferase reporter assay and ChIP-PCR showed that EZH2 was specifically bound to the promoters of GSK-3β and TP53, which are Wnt/β-catenin signaling inhibitors, and that the H3K27me3 repressive marker was also enriched at these promoters, indicating that EZH2 represses the expression of GSK-3β and TP53 (Figure 5). In a previous study, GSK-3β expression was found to be markedly increased after transfection of EZH2 siRNA to promote the proliferation and invasion of ACHN cells via activation of the Wnt/β-catenin signaling pathway [56]. TP53 was first verified as a target gene of EZH2 in cervical cancer. Remarkably, an alteration in p53 regulation has been shown to result in β-catenin protein accumulation in lung cancer [61]. In addition, the immunochemical staining of cervical cancer tissues performed in this study revealed that EZH2 expression was positively correlated with the expression of target genes in the Wnt/β-catenin pathway and that it was negatively correlated with its inhibitors, GSK-3β and TP53 (Figure 6).

**Figure 7:**
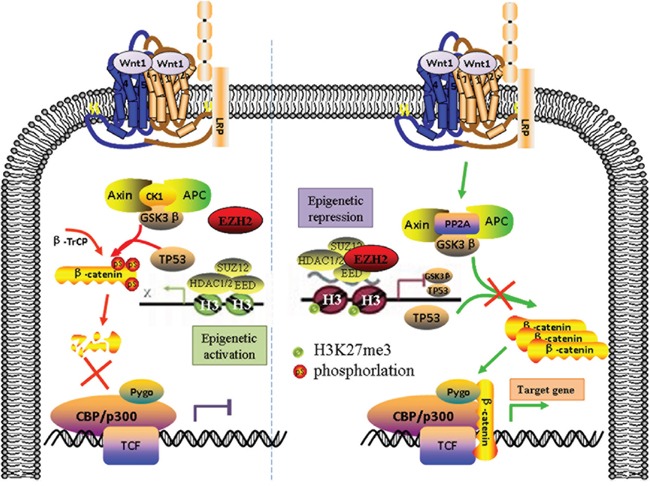
The Schematic diagram of EZH2 activating the Wnt/β-catenin pathway in cervical cancer progression EZH2 promotes cell proliferation and tumor formation in cervical cancer cells by activating the Wnt/β-catenin pathway and reveal a molecular mechanism by which EZH2 mediates the transcriptional repression of GSK-3β and TP53, which are inhibitors of the Wnt/β-catenin pathway

In conclusion, our findings demonstrate that EZH2 promotes cell proliferation and tumor formation in cervical cancer cells by activating the Wnt/β-catenin pathway and reveal a molecular mechanism by which EZH2 mediates the transcriptional repression of GSK-3β and TP53, which are inhibitors of the Wnt/β-catenin pathway (Figure 7).

## MATERIALS AND METHODS

### Cell lines and cell culture

Human cervical cancer cell lines (HeLa, SiHa, C33A, Caski, and HT-3) were purchased from the American Type Culture Collection (ATCC; Manassas, VA). HeLa, SiHa, and C33A cells were cultured in Dulbecco's Modified Eagle's Medium (DMEM; Sigma-Aldrich, St Louis, MO, USA), and CaSki and HT-3 cells were cultured in RPMI-1640 (Sigma-Aldrich) and McCoy's 5A medium (Sigma-Aldrich), respectively, supplemented with 10% heat-inactivated fetal bovine serum (FBS; Invitrogen, Carlsbad, CA, USA). All cell lines were maintained at 37°C with 5% CO_2_.

### Human tissue specimens

A total of 40 normal cervical (NC) tissues, 41 cervical cancer *in situ* (CIS) tissues, and 62 squamous cervical cancer (SCC) tissues were collected from the First Affiliated Hospital of Xi'an Jiaotong University Medical College from 2009 to 2014. All of the procedures followed approved medical ethics practices. None of the patients had received chemotherapy, immunotherapy, or radiotherapy before specimen collection. The histological classifications and clinical staging were in accordance with the International Federation of Gynecology and Obstetrics classification system.

### Immunohistochemistry and immunocytochemistry

Immunohistochemical staining was performed as previously described [62]. EZH2 staining was classified into 2 groups (negative and strong expression) based on the percentage of positive cells and staining intensity [63]. The percentage of positive cells was divided into the following 5 score ranges: <10% (0), 10% to 25% (1), 25% to 50% (2), 50% to 75% (3), and >75% (4). The intensity of staining was classified into the following 4 groups: no staining (0), light brown (1), brown (2), and dark brown (3). EZH2 staining positivity was determined using the following formula: immunoreactivity score (IRS) = intensity score × quantity score. An overall score of ≤3 was defined as negative, >3 as weak positive, and >6 as strong positive. All specimens were evaluated by two pathologists in a blinded manner.

For the immunocytochemistry experiments, cells were cultured on coverslips, fixed with 4% paraformaldehyde for 30 minutes at room temperature, and permeabilized with 0.2% Triton X-100 for 15 minutes at room temperature, and they were then incubated with the primary antibodies described above. An antibody against EZH2 was obtained from Cell Signaling Technology (CST, Beverly, MA, USA), and additional antibodies against Ki67, β-catenin, cyclin D1 and c-myc were obtained from Santa Cruz Biotechnology (Santa Cruz, CA, USA).

### Western blot analysis

Western blot analyses were performed as previously described with lysates from fresh tissues and cells [62]. The primary antibodies used were anti-EZH2 (1:1000, Cell Signaling Technology), anti-GAPDH (1:1000, Santa Cruz Biotechnology), anti-β-catenin (1:1000, Santa Cruz Biotechnology), anti-c-myc (1:500, Santa Cruz Biotechnology), anti-cyclin D1 (1:500, Santa Cruz Biotechnology), anti-GSK-3β (1:1000, Santa Cruz Biotechnology), anti-TP53 (1:500, Santa Cruz Biotechnology), anti-H3K27me3 (1:1000, Cell Signaling Technology), and anti-histone 3 (1:1000, Cell Signaling Technology). The secondary antibodies used included horseradish peroxidase-conjugated anti-rabbit or anti-mouse IgG (Thermo Fisher Scientific, New York, NY, USA).

### Vector construction and transfection

Human full-length EZH2 (NM_152998.2) cDNA was amplified by reverse transcription polymerase chain reaction using mRNA extracted from HeLa cells. The primer sequences were designed as follows:

EZH2-F: 5-′ CCGCTCGAGGCCACCATGGGCCAGACTGGGAAGAAATCTG-3′; and EZH2-R: 5-′ CGCGGATCCTCAAGGGATTTCCATTTCTCTTTCG-3′

The EZH2 DNA fragment was subsequently cloned into the Xho I and BamH I (TaKaRa, Tokyo, Japan) sites of an internal ribosome entry site vector, pIRES2-AcGFP1 (Clontech, Mountain View, CA), to generate the pIRES2-AcGFP1-EZH2 recombinant plasmid.

The pX330-U6-Chimeric_BB-CBh-hSpCas9 plasmid (Plasmid#42230) containing SpCas9 and single guide RNA was obtained from a non-profit plasmid share repository belonging to Feng Zhang (Addgene, Cambridge, MA, USA) [64]. Suitable CRISPR target sites within the positive and negative strands of exon 2 of EZH2 were identified using the ‘CRISPR Design Tool' (http://crispr.mit.edu/) hosted by the Feng Zhang laboratory (Massachusetts Institute of Technology, MA, USA) to obtain the minimum number of off-target sites in the human genome (Supplementary Figure 1A). The respective oligonucleotide pairs were synthetized by GenScript Technologies (Nanjing, China) and were customized to include overhangs compatible for ligation into Plasmid#42230 linearized by digestion with BbsI (New England BioLabs, Ipswich, MA, USA), and these overhangs were located in two places in this vector carrying a guide sequence. Oligonucleotides were annealed and inserted into the plasmid by DNA ligation (TaKaRa, Tokyo, Japan). Then, to prevent screening issues, we structured an additional homologous recombinant plasmid that included the screening marker G418. The two plasmids were co-transfected. Positive clones were verified by Western blot analysis and DNA sequencing of genomic PCR products encompassing the CRISPR target sites (Supplementary Figure 1B and Figure 2A1).

To construct a small interfering RNA expression vector that expressed EZH2-specific short hairpin RNA (shRNA), the following four EZH2 sequences were used, which were obtained from GenePharma Co., Ltd. (Shanghai, China): GCAGCTTTCTGTTCAACTTGATTCAAGAGATCAAGTTGAACAGAAAGCTGCTTTTTTG, GGTAAATCCAAACTGCTATGCTTCAAGAGAGCATAGCAGTTTGGATTTACCTTTTTTG, GCAAAGCTTACACTCCTTTCATTCAAGAGATGAAAGGAGTGTAAGCTTTGCTTTTTTG, and GCTGATGAAGTAAAGAGTATGTTCAAGAGACATACTCTTTACTTCATCAGCTTTTTTG. All transfection experiments were performed using Lipofectamine 2000 reagent (Invitrogen, Carlsbad, CA, USA) according to the manufacturer's instructions. Transfected cells were treated with G418 (Calbiochem, La Jolla, CA, USA) for approximately 3 weeks, and drug-resistant colonies were collected, expanded, and identified.

### Cell growth and cell viability assays

Cells (4×10^4^) were seeded in triplicate with 1.5 ml of media into 35-mm tissue culture dishes for 7 days. The numbers of cells were counted after harvesting every two days using a hemocytometer under light microscopy. Cell viability assays were performed by applying 3-(4,5-dimethylthiazole-yl)-2,5-diphenyl tetrazolium bromide (MTT; Sigma-Aldrich) dye to cells that were seeded in 96-well plates with 1000 cells in each well, as described in a standard protocol. Then, the absorbance at 490 nm was measured (Bio-Rad).

### Flow cytometry analysis

Cell cycle analysis was performed using FACS (Becton Dickinson, Franklin Lakes, NJ) according to the manufacturer's protocol. Cells were harvested and fixed in 75% ethanol overnight at 4°C. Thirty minutes before FACS analysis, the cells were treated with RNase A and were then stained with propidium iodide (Sigma-Aldrich). Cell cycle distribution was analyzed using a FACSCalibur flow cytometer with Mod-Fit LT software.

### Tumor xenograft experiment

The experimental protocols used were evaluated and approved by the Animal Care and Use Committee of the Medical School of Xi'an Jiaotong University. Cells in the exponential growth phase were harvested for inoculation. Tumor cells (1×10^6^) were injected into the subcutises on the dorsa of 4-6 week-old female BALB/c-nude mice (Jackson Laboratories, Inc., Bar Harbor, ME). The tumor volume (V) was determined by the length (a) and width (b) as V=ab^2^/2. The experimental protocols were evaluated and approved by the Animal Care and Use Committee of the Medical School of Xi'an Jiaotong University. At the end of the experiment, the mice were killed by cervical vertebral dislocation, and the weights of the tumors were measured after they were dissected out.

### TOP Flash/FOP-Flash reporter assay

TOP-Flash reporter and pTK-RL plasmids were transiently co-transfected into tumor cells (5×10^4^) in 24-well plates, and the activities of both firefly and Renilla luciferase reporters was determined at 48 hours after transfection using a Dual Luciferase Assay Kit (Promega, Madison, WI, USA) according to the manufacturer's instructions. The TOP-Flash reporter activity is presented as the relative ratio of firefly luciferase activity to Renilla luciferase activity. All experiments were performed three times in triplicate.

### Dual luciferase reporter assay

In brief, plasmids containing firefly luciferase reporters (pGL3 luciferase reporter vectors) and pTK-RL plasmids were co-transfected into cells. Following incubation for 48 h, cell monolayers were harvested by resuspension in passive lysis buffer. Luciferase activity was determined with a luminometer (Promega). The efficiency of transfection was normalized by paired Renilla luciferase activity using a Dual Luciferase Reporter Assay System (Promega). The specific activity was displayed as the fold change of the experimental group versus the control group.

### Quantitative chromatin immunoprecipitation

Quantitative chromatin immunoprecipitation (qChIP) assays were performed using an EZ-ChIPTM Assay Kit (Cat#17– 371; Millipore) according to the manufacturer's protocol. Regions of interest were amplified from precipitated samples by real-time polymerase chain reaction (PCR). Each sample was assayed in triplicate, and the amount of precipitated DNA was calculated as a percentage of the input sample [65]. The primers used in quantitative ChIP assays are listed in Supplementary Table 3.

### Statistical analysis

Statistical analysis was performed with SPSS 18.0 software (SPSS Inc., Chicago, IL, USA). All data were expressed as the group mean ± standard deviation of the mean (SD). A P value of <0.05 was considered statistically significant. For comparison among the groups, the chi-square test or one-way ANOVA was performed.

## SUPPLEMENTARY FIGURES AND TABLES


