# Supplementary material for: EZH2-mediated repression of GSK-3β and TP53 promotes Wnt/β-catenin signaling-dependent cell expansion in cervical carcinoma
Source: Oncotarget. 2016 Apr 15;7(24):36115–29. doi: 10.18632/oncotarget.8741 (PMC5094987; doi:10.18632/oncotarget.8741)
Supplement: Supplementary file 1 [file oncotarget-07-36115-s001.pdf]

# EZH2-mediated repression of GSK-3β and TP53 promotes Wnt/β-catenin signaling-dependent cell expansion in cervical carcinoma

## SUPPLEMENTARY FIGURES AND TABLES

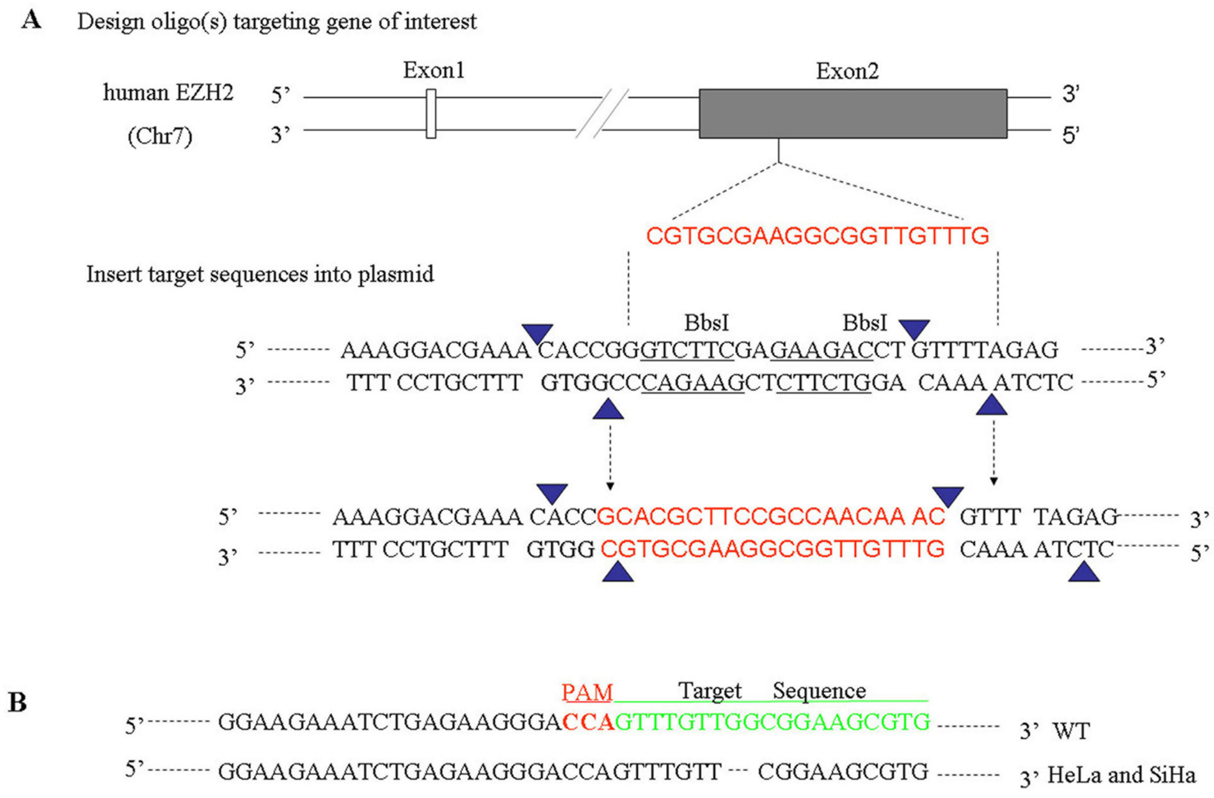


**Supplementary Figure S1: Strategy for generation of human EZH2 knockout clones based on CRISPR/Cas9-system and the generation of EZH2 knockout clones in HeLa and SiHa cell lines. A.** Strategy for generation of human EZH2 knockout clones based on CRISPR/Cas9 mediated editing system. **B.** Detailed insertion/deletion (indel) analysis of EZH2-deficient HeLa and SiHa by DNA-sequencing of genomic PCR products. Consistent with the Western blot analysis, the analysis established the deletion of 2 nucleotides in cell clones.

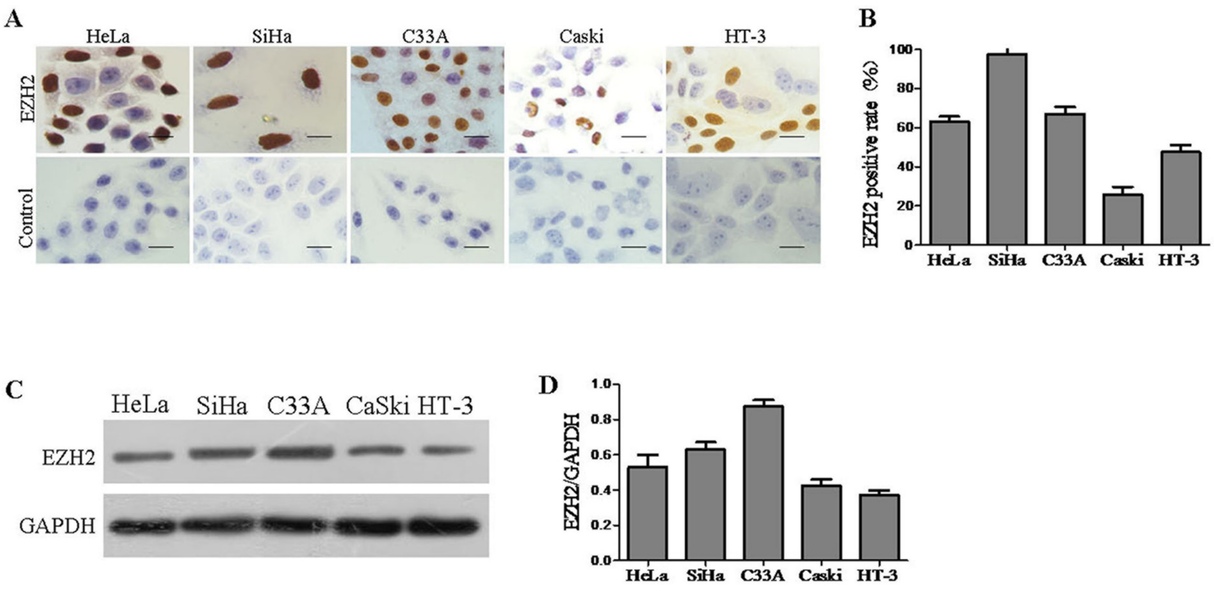


**Supplementary Figure S2: The expression of EZH2 in cervical cancer cell lines. A.** Immunocytochemical staining for EZH2 expression in HeLa, SiHa, C33A, Caski and HT-3 cells, scale bar, 10 μm. **B.** The positive rates of EZH2 expression in cell lines were summarized. **C.** The expression of EZH2 in HeLa, SiHa, C33A, Caski and HT-3 cells was measured by western blot. **D.** The relative expression of EZH2 in HeLa, SiHa, C33A, Caski and HT-3 cells was calculated based on western blot analyses.

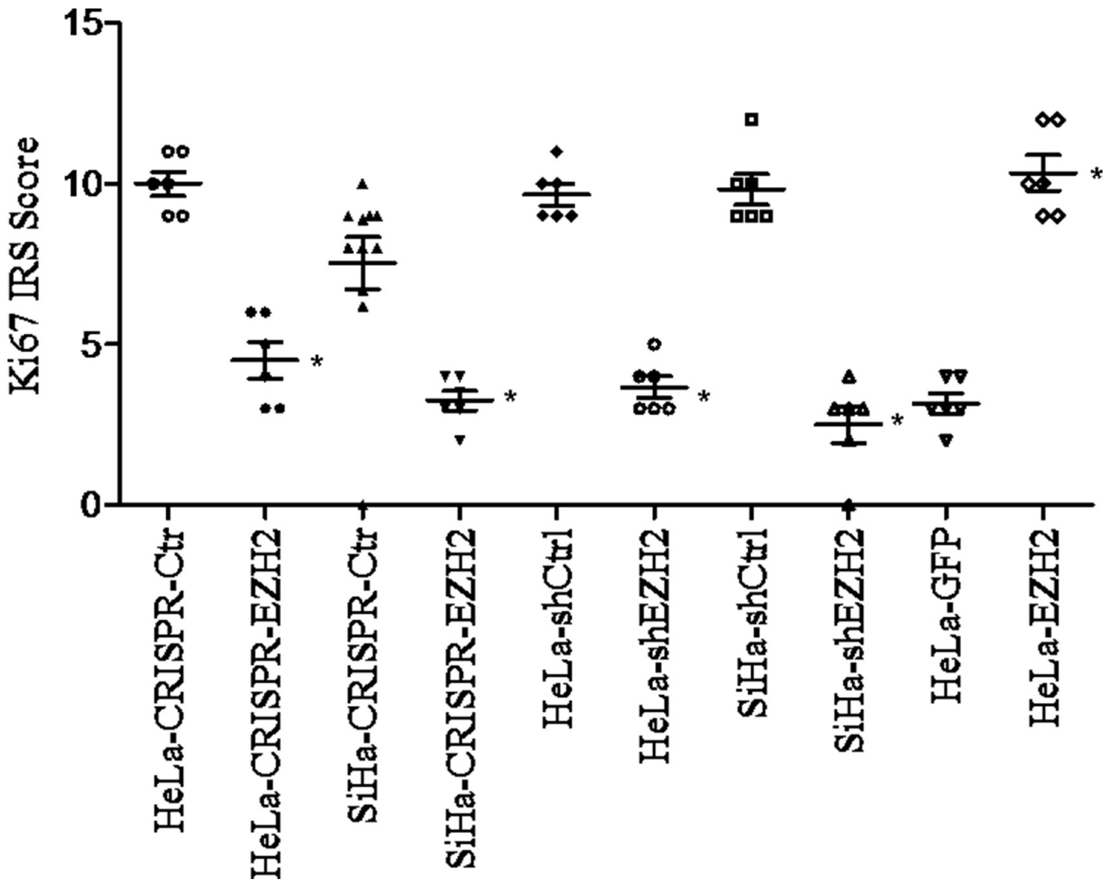


**Supplementary Figure S3: The IHC scores of Ki67 staining in tumor xenografts.** The expression of Ki67 in CRISPR-mediated EZH2-silenced HeLa and SiHa cells, HeLa-shEZH2 and SiHa-shEZH2 cells and EZH2-overexpressing HeLa cells are shown. The values are presented as the mean±SD. * $p<0.05$, ** $p<0.01$.

**Supplementary Table S1: EZH2 Expression in Different Tissue Specimens**

| Specimens | Total | EZH2 Staining | | p |
|---|---|---|---|---|
| | | Negative, N0. (%) | Positive, N0. (%) | |
| Normal | 40 | 35(87.50) | 5(12.50) | |
| Cancer in situ | 41 | 23(56.10) | 18(43.90) | <0.05[a] |
| carcinoma | 62 | 16(25.80) | 46(74.20) | <0.01[b], <0.05[c] |

a: Normal cervix versus cervical cancer in situ.
b: Normal cervix versus carcinoma.
c: Cancer in situ versus carcinoma.

**Supplementary Table S2: List of primer sequences used for luciferase assays in this study, related to Experimental Procedures**

| Name | Sequences |
|---|---|
| TP53-G1-F | CGGGGTACCGACATTTTAACTGATGAGAAGAAAGG |
| TP53-G1-R | CTAGCTAGCTCAGGATTCTCGCCGACCT |
| TP53-G2-F | CGGGGTACCGCCAGGATGGCTTCGAAGTTCTCAG |
| TP53-G2-R | CTAGCTAGCTGGACCGAAATCCCGCGACAGC |
| TP53-G3-F | CGGGGTACCCAGAAAGGCTCCCGTTTGCTTCT |
| TP53-G3-R | CTAGCTAGCTTGTCCCCAGATCCTGTGGCTGG |
| TP53-P1-F | CGGGGTACCGGCACTCAGGAATACAACAATGAAT |
| TP53-P1-R | CTAGCTAGCTTAGGAAGGCTTTCCGTAATATCAC |
| TP53-P2-F | CGGGGTACCACAGCCTTTCAAGAAGTTCTCAGGT |
| TP53-P2-R | CTAGCTAGCGAGATGAAGTGTGAGGTCGATCTGT |
| TP53-F(full-length) | CGGGGTACCCTTACAGCCTTTCAAGAAGTTCTCAG |
| TP53-R(full-length) | CTAGCTAGCGTTTGCTCTCAGCTGGATCCTT |
| GSK-3β-G1-F | CGGGGTACCCTTGGAGAGGGAGGGGAAGTCCT |
| GSK-3β-G1-R | CTAGCTAGCAGAGGCTTGAAGAGTTGAGGACG |
| GSK-3β-G2-F | CGGGGTACCATCTCCCCGCTCCTGGGACCGG |
| GSK-3β-G2-R | CTAGCTAGCGTGGGGAGAAGAAAGCGAAGGGAGG |
| GSK-3β-G3-F | CGGGGTACCGCCCCGCCGAGAGCCTCCTGTGG |
| GSK-3β-G3-R | CTAGCTAGCGTCGTGGCTGACGACCCGACGGT |
| GSK-3β-P1-F | CGGGGTACCTGGAGCTTTGTTCCCATTTTAGCG |
| GSK-3β-P1-R | CTAGCTAGCAGCCAATTCTGCGCGCCCAAGG |
| GSK-3β-P2-F | CGGGGTACCGGCATTTCCCCTCACCTGCT |
| GSK-3β-P2-R | CTAGCTAGCGAAAGGCAGCGCCTTTGGGC |
| GSK-3β-F(full-length) | CGGGGTACCCGCTGCCTTTCTGGAAGCTTTGTTC |
| GSK-3β-R(full-length) | CTAGCTAGCCCTCCCTCTCCAAGGTGGGGAGAAG |

**Supplementary Table S3: List of primer sequences used for ChIP-PCR assays in this study, related to Experimental Procedures**

| Name | Sequences |
| --- | --- |
| TP53-G1-S | CTGATGAGAAGAAAGGATC |
| TP53-G1-A | GGCAGAATTGGTGGAAAT |
| TP53-G2-S | AACCCACCTGTGCTTCC |
| TP53-G2-A | GTGCTAAGGAACACAGTGCT |
| TP53-G3-S | CACTAGGGGAACCAAACTCT |
| TP53-G3-A | TTGTCCCCAGATCCTGTG |
| TP53-P1-S | CGTTTCCATGTACTGAAAGC |
| TP53-P1-A | GAAGGCTTTCCGTAATATC |
| TP53-P2-S | CTTACAGCCTTTCAAGAAGT |
| TP53-P2-A | AGGTTTATTGTCCCCCATC |
| TP53-S(3’UTR) | CCACTTCTTGTTCCCCACT |
| TP53-A(3’UTR) | GGACAAAGCAAATGGAAGT |
| GSK-3β-G1-S | ACCTTGGAGAGGGAGG |
| GSK-3β-G1-A | CGCTTTGTGATTGGCT |
| GSK-3β-G2-S | CCGAAGGCCAATCACAC |
| GSK-3β-G2-A | GCAAAAGGGAGAGGGAG |
| GSK-3β-G3-S | AGAATTGGCTGCGCC |
| GSK-3β-G3-A | TCGGTGCAGCAAATCG |
| GSK-3β-P1-S | TCCGTCTGGCCCTTTC |
| GSK-3β-P1-A | TTCTGCGCGCCCAAG |
| GSK-3β-P2-S | CATGGGCGTTTCCAGAG |
| GSK-3β-P2-A | GAAAGGCAGCGCCTT |
| GSK-3β-S((3’UTR) | GGGAGGTCAATAAAGTTTGG |
| GSK-3β-A((3’UTR) | TGGCAGCCTCCTGTACAAG |
